# Slow release anti-fungal skin formulations based on citric acid intercalated layered double hydroxides nanohybrids

**DOI:** 10.1186/s13065-015-0106-3

**Published:** 2015-05-21

**Authors:** Jayoda Perera, Manjula Weerasekera, Nilwala Kottegoda

**Affiliations:** Department of Chemistry, Faculty of Applied Science, University of Sri Jayewardenepura, Nugegoda, Sri Lanka; Sri Lanka Institute of Nanotechnology, Center for Excellence in Nanotechnology, Nanotechnology and Science Park, Pitipana, Homagama Sri Lanka; Department of Microbiology, Faculty of Medical Science, University of Sri Jayewardenepura, Nugegoda, Sri Lanka

**Keywords:** Layered double hydroxides, Citric acid, Nanohybrid, Anti-fungal, *Candida species*, Slow release

## Abstract

**Background:**

During the past few decades, the occurrence of superficial fungal infections has rapidly increased. As the fungal infections take longer time to get cured, concepts such as designing drugs with extended persistence and controlled release have gained attention. In this context, nanotechnology has been identified as the latest technological revolution which has opened up new pathways for designing new therapeutic materials. Out of the many available nano-structures layered double hydroxides have gained increased scientific attention in applications as slow and controlled release drug formulations. This study focuses on the encapsulation of citric acid which has anti-fungal properties into a Mg-Al- layered double hydroxide (LDH) in order to be used as slow release topical skin formulations.

**Results:**

Citrate ions were encapsulated into Mg-Al LDH using one step co-precipitation reaction. The successful intercalation of citrate ions into the layered structure has been proved referring to the expansion in the interlayer spacing as observed by the shift in the basal peak of the powder X-ray diffraction pattern. Fourier transform infra-red spectroscopy data suggests the change in the electron density around the carboxylate groups of the citrate ion thus providing evidences for formation of encapsulated hybrid composite. The resulting nanohybrid has been then, introduced into a general body cream formulation containing cocoa-butter. Both citrate LDH and the resulting body cream formulations demonstrated prolonged slow release characteristics up to 8 h in aqueous medium under different pH values (3, 4, and 5) compared to quick and fast release of pure citric acid. It was observed that the slow reelase was most efficient at low pH values. The encapsulation between the nano-layers and citrate ions are the key to the slow release characteristics. The body cream has been tested for the anti-fungal activity against three common *Candida species (C. albicans, C. glabrata, C. tropicalis)*. The novel nanohybrid has shown an improved activity and slow release characteristics up to 48 h against the *C. albicans* and *C. glabrata* but not for *C. tropicalis*.

**Conclusion:**

The study confirms that the citrate ion intercalated LDHs have the potential for use in future slow release antifungal drug formulation.

**Graphical Abstract:**
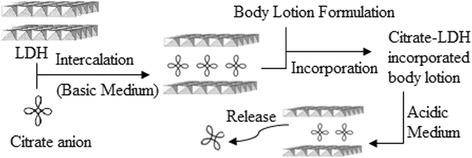
Slow release nanohybrids based on citrate intercalated layered double hydroxides

## Background

*Candida species* are the most common cause of fungal infections [1]. *Candida species* produce diseases that range from non-life-threatening superficial to systemic infections that may involve virtually any organ [2]. As it can cause broad range of infections it requires a broad range of therapeutic strategies.

Over the past few decades the word ‘nano’ has become rapidly insinuating into the world consciousness. It has already conjured up speculation about a seismic shift in almost every aspect of science and engineering including the advancements in medical sciences. This technology involves the use of particles or materials that have at least one dimension in the range of 1–100 nm size scale. In this size scale, unexpected properties of the material are derived by the resulted high surface area and the quantum mechanical effects. As a result, both chemical and physical properties of these materials are much more different compared to the bulk material.

Nanotechnology has already shown potential in pharmaceutical industry [3]. It can be helpful from developing antibiotic drug to more wider and complex applications such as use of DNA – nanoparticle hybrid to store genetic information [4]. This technology becomes important in novel pharmaceutical approaches in designing effective and efficient drug delivering system. It has been demonstrated that the applications of nanomaterials, such as carbon nanotubes, silica nanowires and gold nanoparticles have increased the stability and durability of the drugs [5]. The enhanced activity of nanoparticles as drug delivery systems has broadened the application of nanotechnology in antitumor drug design [5].

There is also an increased scientific interest in exploring the potential of layered nanomaterials in slow and controlled release drug delivery applications. In this backdrop, layered double hydroxides (LDHs) which are a class of clays, composed of brucite like nanolayers with a thickness of 0.48 nm, and interlayer charge balancing anions have gained a specific attention. Interestingly, these interlayer anions are able to undergo ion exchange reactions thus, allowing to engineer a range of layered materials with preferred properties. For example, Li *et al.* intercalated Fenbufen as anti- inflammatory drug, into the LDH matrix with a coating of enteric polymers to be used in the digestive tract as a slow release drug formulation [6]. Samindra and Kottegoda have reported the successful intercalation of natural antibacterial curcumin , into Mg: Al (2:1 ratio) LDH and demonstrated its slow release behavior [7]. Latip *et al.* intercalated anionic ciprofloxacin, which is a broad spectrum antibiotic compound in therapeutic purpose, into Zn Al- LDH and the sustained release and the enhanced toxicity effect were studied [8]. Furthermore, there had been several successful attempts of using LDHs in order to intercalate antibacterial compounds especially in approaches of pharmaceutical, cosmetic and agricultural industries. Bugatti *et al.* has intercalated 2,4-dichlorobenzoate, benzoate and para- hydroxybenzoate which are commonly used as antimicrobial agents in food packaging purposes, into the Zn Al- LDH via anion exchange reaction [9]. Antibacterial activity and enhanced release behavior of these LDHs have been studied and confirmed.

In this study, attempts are made to encapsulate citrate anions into an LDH matrix and to study its potential as a slow release antifungal agent in skin topical applications.

## Results and discussion

### Characterisation of citrate-LDH

**Fig. 1:**
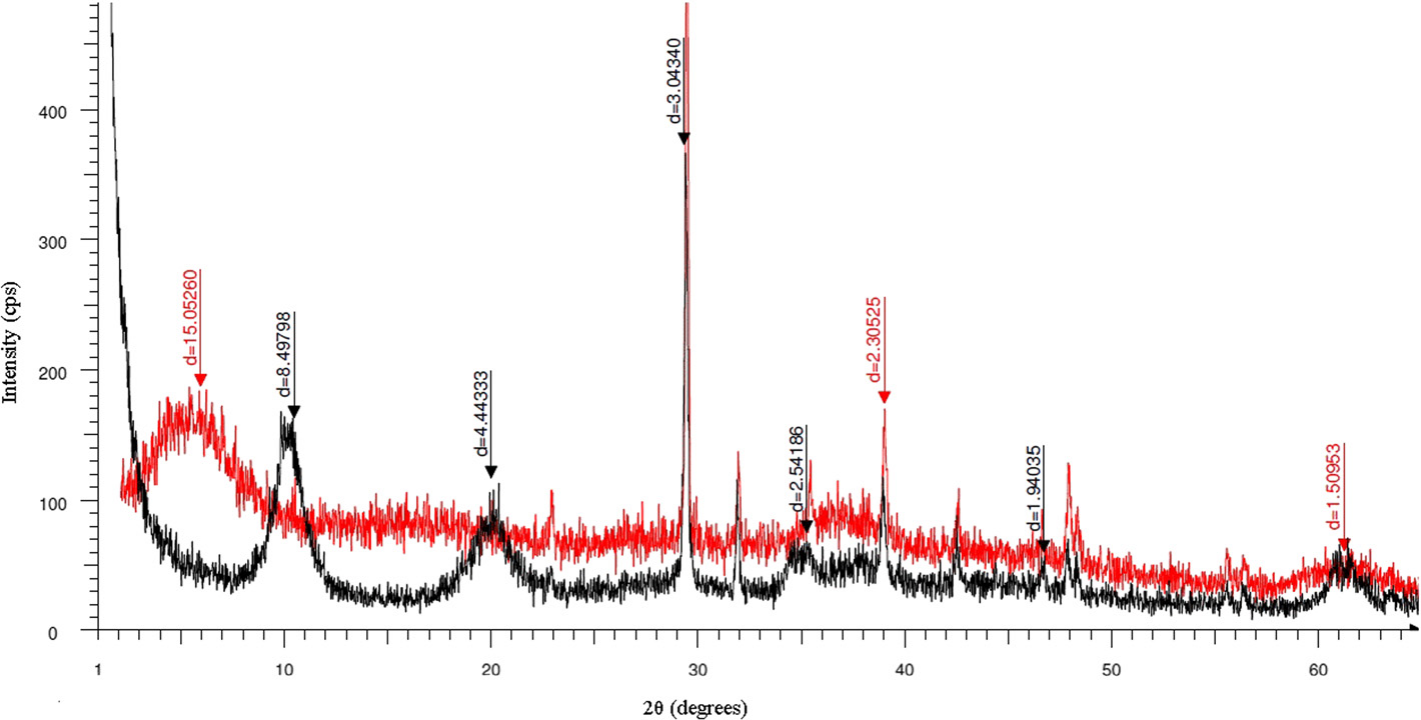
PXRD patterns of nitrate – LDH (black) citrate – LDH (red) PXRD patterns were recorded on the dried (100 °C) white powder obtained by *insitu* co-precipitation reactions

PXRD technique was used tounderstand the successful intercalation of citrate ions into the LDHs (Fig. 1).

The PXRD pattern of pure nitrate – LDH agrees well with that reported in literature [6]. Based on a hexagonal unit cell the PXRD peaks were indexed resulting in unit cell parameters of *c* = 2.52 nm and *a* = 0.27 nm. Considering the basal reflections an inter-layer spacing of 0.87 nm leading to a gallery height of 0.39 nm is suggested and the observed value agrees with previous results (0.88 nm) [6]. This gallery height suggests a vertical arrangement of nitrate anions within the layers, considering the size of nitrate ions as 0.4 nm [10].

**Fig. 2:**
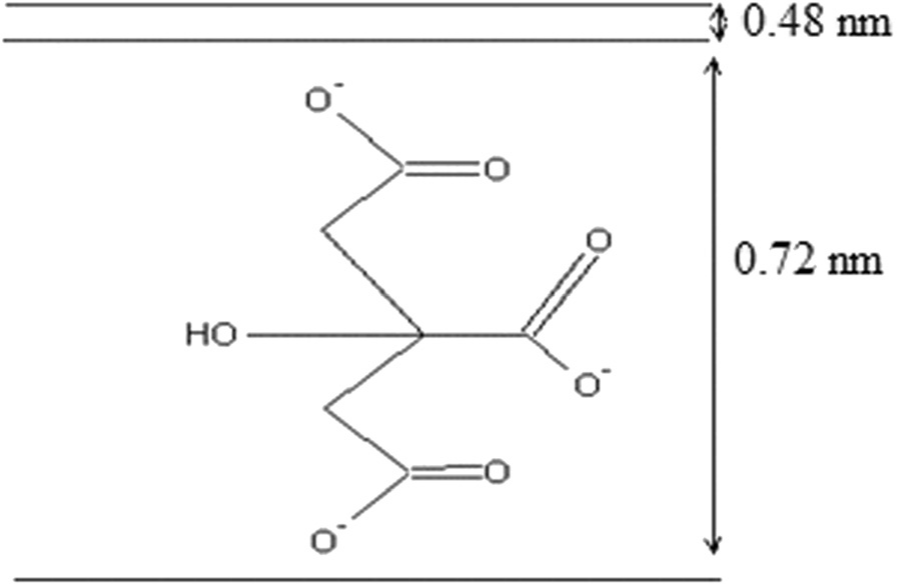
Interlayer arrangement of citrate ion within the layers of Mg-Al LDH. The inter-layer arrangement was derived based on the basal spacing observed for the citrate LDH (1.20 nm) and the theoretical molecular size of citrate ion (0.72 nm)

When compared with the standard nitrate – LDH, the PXRD pattern of citrate–LDH demonstrates a lower wave number shift for the basal peaks (*003* and *006*), while there is no noticeable change in the non-basal reflections. This suggests that there is an increase in the interlayer spacing after intercalation of citrate thus the *c* parameter of the unit cell while there is no effect to the *a* parameter thus suggesting a constant inter-metallic distance. The observed interlayer spacing for citrate–LDH is 1.5 nm. Given that the thickness of the brucite-like layer of LDH is 0.48 nm, the gallery height of 1.02 nm is observed. Considering the size of citrate ion as 0.72 nm [11], vertical monolayer arrangement can be suggested within the interlayer region (Fig. 2).

The absence of any peaks arising from nitrate- LDH confirms that there is no nitrate intercalation in citrate–LDH due to anionic competition during the synthesis. The observation could be explained referring to the preferences of anionic valency during formation of LDHs. Generally, higher valent anions are preferred over lower valent anions due to the lower steric hindrance that arise within the interlayer spaces. In addition citrate ions create a very strong hydrogen bond network within the interlayer region due to the presence of OH and carboxylate groups.

**Fig. 3:**
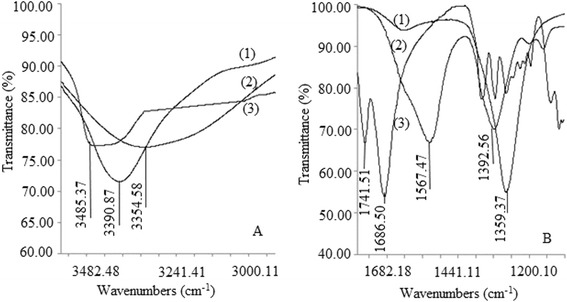
FT-IR spectra for **a** hydroxyl **b** carbonyl stretching regions of (1) nitrate-LDH (2) citrate–LDH (3) pure citrate

The nature of the bonding environment of citrate ions within the layers were studied referring to shifts of hydroxyl and carbonyl peaks of citrate ions in FTIR spectra (Fig. 3).

Broad and intense peak is observed at 3485 cm^−1^ for pure citric acid which is assigned to the hydroxyl stretching frequency. Sharp intense peak at 1686 cm^−1^ for pure citric acid is due to the carboxyl stretching modes. For nitrate–LDH, the broad peak at 3390 cm^−1^ is due to the hydroxyl stretching modes of the layered hydroxyl groups and the peak at 1359 cm^−1^ is assigned for the nitrate group stretching frequency which present within the layers. Intercalation leads to the formation of strong H bonding network. Therefore, peak broadening can be observed in nitrate–LDH than in pure citric acid.

Intense broad peak is observed at 3354 cm^−1^ for citrate–LDH corresponding to the hydroxyl and water stretching frequencies. These lower wave number shift from the pure citrate ions and nitrate-LDH suggest the increased electron density around the functional groups. The strong H-bonding network is responsible for the improved electron density around OH groups. When compared with the nitrate-LDH, for citrate–LDH anions has the ability to form stronger H-bonding network than the nitrate groups due to the presence of larger number carboxylate and hydroxyl groups. This leads to the peak broadening in citrate–LDH spectra. Sharp peaks observed at 1567 cm^−1^ and 1386 cm^−1^ for citrate–LDH correspond to the asymmetric and symmetric stretching modes of the carboxylate groups. The stretching vibrations of the carboxylate group of pure citric acid at 1686 cm^−1^ has been shifted to 1350 – 1600 cm^−1^ after the intercalation into layers. That is due to the stronger intermolecular interactions such as H- bonding and Van Der – Waals interactions, experienced by the intercalated citrate anions within the matrix. Those interactions stabilize the system and decrease the vibration modes of the molecule.

Nitrate stretching peak at 1359 cm^−1^ is absent in citrate–LDH confirming that the formed LDH does not contain any intercalated nitrate ions. The FTIR characterization verifies the successful synthesis of citrate–LDH nanohybrid.

**Fig. 4:**
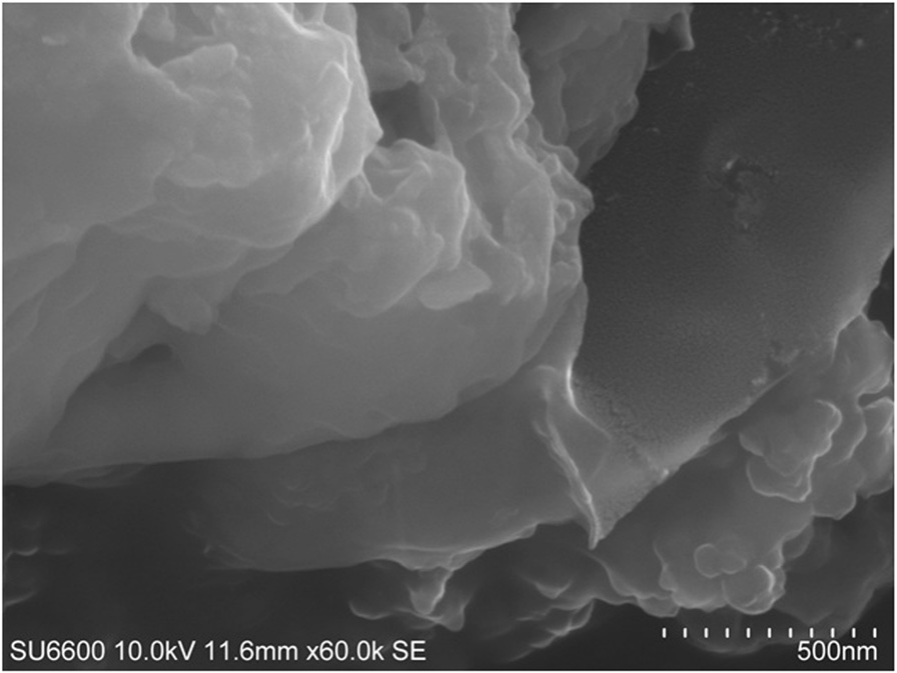
SEM image of citrate-LDH. The internal morphologial features of the citrate-LDH is demonstrated in this image. The images have been captured in the secondary electron mode with a magnification of 60,000 and an accelerating voltage of 10 k eV

The morphological features of the citrate-LDH was studied using scanning electron microscopic imaging, Fig. 4. As evidenced by the image the composite demonstrated a typical layered structure containing nanometer size thick platelets. The crystallinity of the LDH was low according to the SEM images further corroborating the PXRD data. EDX data suggested a Mg: Al ratio of 2 as expected.

### Release behavior of citrate-LDH and the body lotion at different pH values

Release behavior of the citrate-LDH, pure citric acid were compared at different pH values (3, 4, 5) while that was repeated for citrate-LDH intercalated body lotion at pH 4 which is the skin pH under *Candida species* infections.

**Fig. 5:**
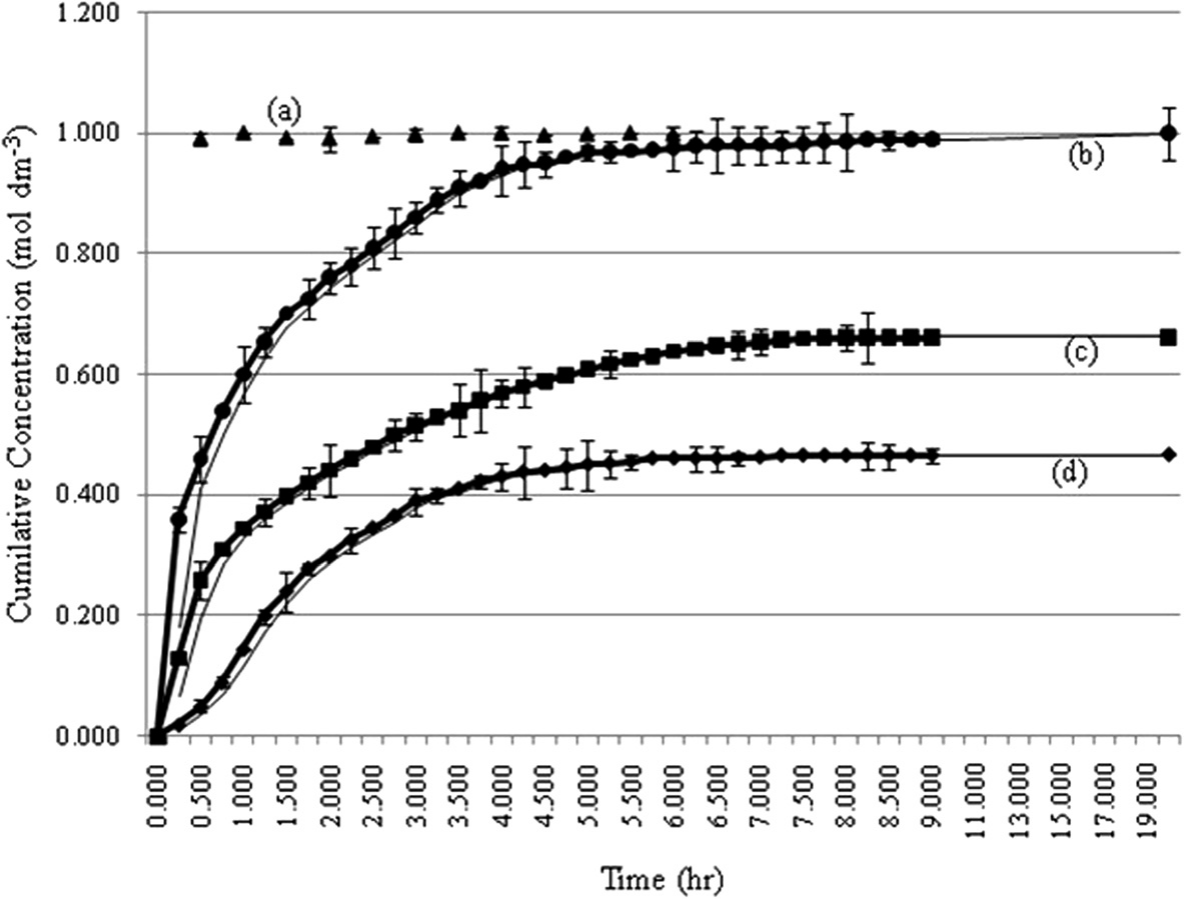
Release behavior of **a** citric acid (pH 5), citrate-LDH at pH **b** 3, **c** 4 and **d** 5

As shown in Fig. 5 citrate–LDH shows a gradual release of intercalated citrate ions into the medium over a considerable time period. Within the first eight hours citrate is released in a slow and sustainable manner while a constant releasing pattern is observed afterwards. On the other hand, pure citrate shows a rapid releasing pattern, where all the added citrate ions were released within the first hour. This observation confirms the slow releasing ability of citrate–LDH is a result of the layered matrix of LDH.

Furthermore, the amount of citrate released at pH 3 is higher than that of pH 4 and pH 5. In the acidic medium protons attack to the citrate ions and thereby citrate ions get protonated and released to the medium thus leading to higher amount of citrate ions to release from the layered matrix to the medium.

**Fig. 6:**
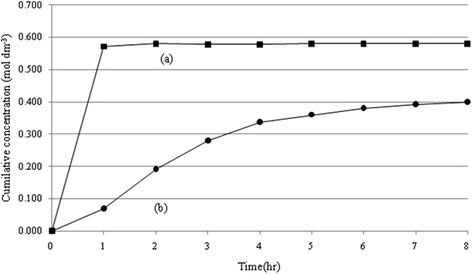
Release behavior of the **a** citrate ion incorporated body lotion **b** citrate-LDH incorporated body lotion

The release behavior of the citrate-LDH incorporated body lotion is compared with that of citrate incorporated body lotion at pH 4 which is closer to the skin pH under fungal infections (Fig. 6). According to the above result it is confirmed, that the citrate–LDH incorporated lotion also shows a similar slow release pattern of intercalated citrate ions into the medium to the citrate-LDH, over a considerable time period. However, only 40 % of the intercalated citrate is released within the first eight hours. That limitation to the release of citrate may be due to the effect from the lotion matrix. This observation provides evidence of the potential of citrate-LDHs to be used as slow release antifungal formulations in topical dermatological applications.

### Antimicrobial properties against *Candida species*

The antifungal activities of pure citrate, citrate–LDH and citrate–LDH incorporated lotion were tested against the growth of *Candida albicans, Candida tropicalis* and *Candida glabrata.* Organic acids are widely used as buffer agents in medical solutions as well as the preservatives in food [12]. In addition to their inhibitory effect on the growth of food spoilage microorganisms, organic acids were shown antibacterial and antifungal activity against various infectious pathogens [13]. These organic acids may have several inhibitory mechanisms. It may, cause the reduction of interior pH of microbial cells or depression of proton motive force [12]. It may also prevent the production of malic acid, which is an important intermediate in the cellular respiration, thereby reduce the energy production of microorganisms and inhibit their growth [13].

According to the observations in the study; pure citrate, citrate–LDH and citrate–LDH incorporated lotion shows inhibitive activity against all three strains of Candida*.* When compared with the pure citric acid, incorporated citrate is able to inhibit the growth of fungi for longer time period (Table 1). Since the lotion matrix does not shows any activity against the growth of the fungi, the inhibitive action of the citrate–LDH incorporated lotion is approached only by the incorporated citrate ions.

**Table 1:** Inhibitory activity of test compounds against *C. albicans, C. glabrata* and *C. tropicalis*

Substances tested	Visually inspected growth					
	*Candida albicans*		*Candida glabrata*		*Candida tropicalis*	
	After 24 h	After 48 h	After 24 h	After 48 h	After 24 h	After 48 h
Fluconazole	0	0	0	0	0	+1
Pure citric acid	0	+2	0	+2	0	+2
Citrate-LDH	0	0	0	0	0	+1
Body lotion matrix	+1	+2	+2	+2	+1	+2
Citrate-LDH incorporated lotion	0	0	0	0	0	+1

The study of slow release antifungal activity is based on visual observation of the fungal growth in a Muller Hinton culture media and the criteria used is given below

+2: Growth is equals to that of the negative control

+1: Growth approximately 50 % that of the negative control

0: No visible growth at all

Although *C. albicans* and *C. glabrata* express the similar growth response towards the tested substances, behaviour of the *C. tropicalis* towards the same substances after 48 h is altered. That type strain of C*andida sp.* may have the ability to adjust to the changes in the growth medium made by the organic acid. Therefore, the inhibitory activity of citric acid and intercalated citrate is efficient towards the growth of *C. albicans* and *C. glabrata* but not that efficient towards *C. tropicalis.*

#### Synthesis of LDHs

Nitrate and citrate intercalated Mg-Al-LDHs (Mg:Al 2:1) were prepared. Nitrate intercalated LDH (nitrate=LDH) was prepared as explained by Li *et al*. [6] and was used as the standard for the comparison purposes. Citrate intercalated LDH (citrate-LDH) was synthesized to explore its potential as a slow release anti-microbial compound in skin formulations.

A solution containing 0.25 mol of Mg (NO_3_) _2_.6H_2_O and 0.125 mol of Al (NO_3_) _3_.9H_2_O in 250.00 cm^3^ of distilled water was added drop wise to a solution containing 0.25 mol of NaNO_3_ in 100.00 cm^3^ of distilled water, under an inert atmosphere and vigorous stirring condition at 60 °C. The pH of the solution was maintained at 10 throughout the experiment using a solution of 2.0 mol dm^−3^NaOH. Then, the solution was aged at 60 °C for 14 h and the precipitate obtained, was separated by filtration. Then, the precipitate was washed thoroughly with distilled water until excess NaOH get removed and dried at 80 °C for 18 h resulting in nitrate intercalated LDH.

Citrate intercalated LDH was prepared using a similar procedure as explained for nitrate-LDH. Instead of 2.5 moldm^−3^of NaNO_3_, 2.55 mol dm^−3^ of tri sodium citrate solution was used as the anion to be intercalated.

### Experimental

All chemicals and reagents used for the experiments were of analytical grade and distilled water was used for the experimental work.

#### Incorporation of citrate anions and citrate=LDH into a body lotion formulation

Citrate anion and citrate-LDH were incorporated into a body lotion formulation as explained below.

A mixture containing distilled water and glycerin (2:1 ratio) was heated for 20 min and added to a mixture containing unrefined pure cocoa butter and emulsifier (1:2 ratio), while continues homogenizing. Then, the mixture was cooled down to room temperature and 1.00 g of tri sodium citrate was added and homogenized for 1 h. A similar procedure was followed to introduce citrate-LDH in to the body lotion formulation.

#### Release behavior studies

Release behavior studies of the citrate-LDH (1.00 g of citric acid), and pure citric acid (1.00 g) were performed using a dialysis membrane bag containing a magnetic stirrer, dipped in a receptor compartment containing 20.00 mL of phosphate buffer (pH 3, 4,5). The receptor solution was stirred at room temperature and at suitable time intervals the releasing medium (5.00 mL) was collected. The amount of released citrate was measured and quantified by UV- Visible spectrophotometer. Release behavior studies of the pure citrate (equivalent weight is 0.632 g) and citrate-LDH incorporated lotion were carried out using the same method with the receptor solution at pH 4.

#### Determination of antifungal activity

##### Preparation of sample

The antifungal activity against three Candida species (*Candida albicans* (ATCC 90028), *Candida tropicalis* (ATCC 13803)*,* and *Candida glabrata* (ATCC 90030)) of the nanohybrid formulation was compared with that against citrate and fluconazole, one of the common topical antifungal administration.

Solutions of 0.25 g dm^−3^ citric acid, citrate-LDH and fluconazole were prepared using normal saline and the solutions were autoclaved before the experiments.

##### Preparation of agar medium for pour plate method

Mueller- Hinton agar (Oxoid, England) medium was prepared and after autoclave the media was left to semi cool in a sterilized environment [14]. Pre prepared samples (2.00 mL of each) were placed in separate sterile glass plates and 18.00 mL of prepared agar medium was then poured into plates and swirl clockwise and anticlockwise and allowed to solidify.

##### Preparation of inoculum

Three of type strains of *Candida species* (*Candida albicans* (ATCC 90028), *Candida tropicalis* (ATCC 13803)*,* and *Candida glabrata* (ATCC 90030)) were used to study the antifungal activity. Young (24 h old) and pure cultures of type strains were inoculated in Muller – Hinton agar (Oxoid, England) plates and incubated at 37 °C for 24 h [14]. Then, inoculums were prepared by dissolving loop-full of each species in sterile normal saline and the turbidity of suspension was adjusted to 0.5 McFarland standard.

##### Inoculation and incubation of the medium

Inoculation and incubation of the medium was carried out as explained by Nadeem and co-workers [15]. Each prepared inoculums of *C. albicans, C. tropicalis* and *C. glabrata* were then separately spotted (40.00 μL) on the prepared culture plates. All the experiments were carried out in triplicates. Fluconazole was used as the standard laboratory control while sterile distilled water was used as a negative control for the experiment. The inoculated plates were incubated at 37 °C aerobically in an incubator and fungal growth in each plate was observed after 24 h and 48 h.

### Characterization

Powder X-Ray Diffraction (PXRD) characterization was carried out to identify the crystalline phases in the synthesized LDHs. Brucker D8 focus X-ray powder diffraction meter was used to analyze the powdered LDHs using Cu Kα radiation (wave length – 1.540 Å) over 2θ angle, of 2^0^ to 70^0^ with a step size of 0.02^0^.

Fourier Transform Infra-Red (FTIR) spectroscopy technique was used to identify functional groups in the synthesized materials. Nicolet IS 10 instrument was used to analyze the powdered sample using diffuse reflection mode in the range from 600 cm^−1^ to 4000 cm^−1^. The sample was mixed with potassium bromide in 1:100 ratio and then the mixture was ground to a fine powder. Then, a disc having an even surface was prepared by compressing the powdered sample.

Thermo Gravimetric Analysis (TGA) was used to study the weight loss of the material as the function of the temperature allowing to understand the thermal stability of the synthesized materials. SDTQ 600 thermo gravimetric analyzer was used in this study. The sample (10 mg) was heated at a rate of 10 °C per minute in a nitrogen atmosphere in a temperature range of 30–1000 °C. Q series 600 software was used to this analysis.

UV- 2602 Labomed, Inc. single beam scanning spectrophotometer was used for citric acid release studies as a function of time at different pH values.

Morphological studies of the citrate LDH was carried out using scanning electron microscopy (SEM) SU6600, in secondary electron mode. Energy dispersive X-ray analysis (EDX) was carried out using the EDX detector attached to the SU6600 SEM.

## Conclusion

Mg-Al-citrate-LDH has been successfully synthesized and introduced into a body cream formulation containing cocoa-butter. The resulting nanohybrid body lotion demonstrated slow release characteristics for citrate anion at acidic pH values. Improved and prolonged activity of the novel nanohybrid has been proved against *Candida albicans* and *Candida glabrata* compared to fluconazole, one of the common topical antifungal drug.
